# Multiple-Cumulant-Matrices-Based Method for Exact NF Polarization Localization with COLD Array

**DOI:** 10.3390/s25103244

**Published:** 2025-05-21

**Authors:** Jiefeng Zheng, Haifen Meng, Zhuang Luo, Huayue Wu, Weiyue Liu, Hua Chen

**Affiliations:** 1 Faculty of Electrical Engineering and Computer Science, Ningbo University, Ningbo 315211, China; zhengjf7663@163.com (J.Z.); 19807857024@163.com (H.M.); liuweiyue@nbu.edu.cn (W.L.); dkchenhua0714@hotmail.com (H.C.); 2 School of Materials Science and Chemical Engineering, Ningbo University, Ningbo 315211, China; 236003061@nbu.edu.cn

**Keywords:** spherical wavefront, fourth order cumulant, ESPRIT, asymptotic variance

## Abstract

As a key technology for the fifth-generation of mobile communications, massive MIMO systems enable massive user access via large-scale arrays. However, their dense deployment extends the near-field (NF) region, introducing new localization complexities. Based on an exact spherical wavefront model, this paper proposes a multiple-cumulant-matrices-based method for NF source localization using a Co-centered Orthogonal Loop and Dipole (COLD) array. Firstly, following the physical numbering of array elements, we can construct multiple polarization cumulant matrices, which can then be cascaded into a long matrix. Next, the signal subspace can be obtained through eigen-decomposition of this long matrix, from which the horizontal and vertical components can be further separated. By applying ESPRIT, joint angle, range, and polarization parameters can be estimated. In addition, the asymptotic variances for joint spatial and polarization parameters are analyzed. Compared with existing NF polarization algorithms, the proposed method exhibits better parameter estimation and is consistent with a theoretical asymptotic performance.

## 1. Introduction

In array signal processing, source localization is an important problem, with widespread applications in wireless communication and radar systems [1,2,3,4,5,6,7]. In practical scenarios, such as multiple-input multiple-output (MIMO) systems equipped with a large antenna array, the target source crosses the Fresnel boundary, entering the NF region. When the sources move from the far-field (FF) region, the planar wavefront assumption is not available, requiring characterization by a spherical wavefront that is bivariate in both the Direction-of-Arrival (DOA) and range parameters, and the propagation attenuation induces range-dependent amplitude variations among sensors, thereby rendering FF localization methods ineffective.

Nowadays, numerous algorithms for FF localization have been successfully generalized for NF applications, such as the maximum-likelihood (ML) method [8], second-order statistics (SOS) methods [9,10,11,12] and fourth-order cumulant (FOC) methods [13,14,15]. Based on the wavefront approximation principle, i.e., Fresnel approximation, these methods suffer from amplitude and phase loss compared with practical wireless environments [16,17,18]. For amplitude attenuation, several works have addressed this issue. In [19], a delay-correlation SOS-based method was proposed considering amplitude attenuation; however, to satisfy the symmetric property of the array, this method utilizes an approximation phase item. Recently, based on an exact spherical wavefront model, Ref. [20] proposed a delay FOC-based method, which can achieve underdetermined estimation by using a cumulant matrix and vectorization operation, but it requires 3-D spectral search, suffering from an extremely high complexity. In [21], a mixed FF and NF localization method was proposed using coarse and fine estimation from the amplitude and phase item, and this method is closed-from, but suffers from aperture loss.

Although all the abovementioned algorithms were designed for effective spatial parameter estimation, they neglect polarization information, which is a fundamental feature of electromagnetic sources. With the introduction of polarization-sensitive arrays, both spatial and polarization parameter localization are possible. In [22], a generalized ESPRIT method was performed for NF polarization source localization based on a cross-dipole array, which can decouple spatial and polarization parameters, but it can only estimate spatial parameters. In [23], a rank-reduction-based method was proposed, but it still required a 2-D spectral search to estimate polarization parameters. The aforementioned methods [8,9,10,11,13,15,22,23] employed Fresnel approximation. Then, a COLD array-based exact NF polarization localization method was proposed in [24], which can estimate the polarization rotational matrix, but estimates spatial parameters from range-dependent amplitude attenuation, resulting in a lower estimation accuracy.

To address the issue of exact NF polarization source localization, by employing a COLD array, this paper proposes a multiple-cumulant-based DOA estimation method. Cumulant matrices related to various array elements are calculated, which can then be combined in a long matrix, avoiding the aperture loss. By using ESPRIT, the proposed method can achieve spatial and polarization decoupling and joint estimation of four-dimensional (4-D) parameters. Moreover, joint spatial and polarization parameter performance evaluation was performed to demonstrate the algorithm’s validity.This paper represents an original contribution in three aspects:

(1) Unlike conventional NF polarization localization methods, the proposed algorithm completely avoids systematic errors induced by model approximations.

(2) Multiple cumulant matrices are constructed and cascaded into a long matrix, so the manifold matrix for joint parameters can be estimated without aperture loss.

(3) We derive an analytical expression for the asymptotic variance under an exact NF polarization condition.

## 2. Signal Model

**Figure 1:**
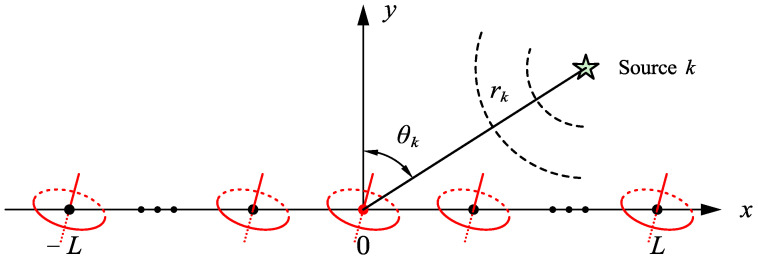
COLD array configuration for exact NF source localization.

As depicted in Figure 1, assume that there is a linear array deployed along the *x*-axis consisting of 2L+1 COLD antennas, where the center antenna, i.e., the 0-th antenna, serves as amplitude-phase reference. Consider *K* narrowband completely polarized signals impinging on the array from $\left(\theta_{1},r_{1}\right),\cdots ,\left(\theta_{k},r_{k}\right),\cdots \left(\theta_{K},r_{K}\right)$ with corresponding polarization parameters $\left(\gamma_{1},\eta_{1}\right),\cdots ,\left(\gamma_{k},\eta_{k}\right),\cdots ,\left(\gamma_{K},\eta_{K}\right)$, where $\theta_{k}$, $r_{k}$, $\gamma_{k}$ and $\eta_{k}$ represent the angle, range, polarization angle, and polarization phase difference of the *k*-th signal $s_{k}\left(t\right)$, respectively.

Based on spherical wavefront geometry, the range between the *k*-th source and *l*-th sensor is given by

(1) $$r_{l,k}=\sqrt{r_{k}^{2}+d_{l}^{2}-2r_{k}d_{l}\sin\theta_{k}},$$

where $d_{l}$ represents the distance between the *l*-th COLD and reference point, l=−L,...,0,...,L. Therefore, the exact spatial amplitude-phase corresponding to between *k*-th source and *l*-th sensor be expressed as

(2) $$b_{l,k}=\frac{r_{k}}{r_{l,k}}e^{j\frac{2\pi }{\lambda }\left(r_{k}-r_{l,k}\right)}=\rho_{l,k}e^{j\tau_{l,k}},$$

where $\rho_{l,k}$ stands for the amplitude attenuation, $\tau_{l,k}=\frac{2\pi }{\lambda }\left(r_{k}-r_{l,k}\right)$ stands for the exact phase shift caused by propagation delay, and then we can obtain the spatial steering vector of the *k*-th source, which can be expressed as

(3) $$b\left(\theta_{k},r_{k}\right)={\left[b_{-L,k},...,b_{0,k},...,b_{L,k}\right]}^{T}.$$

Additionally, for the COLD array, the polarization steering vector of the *k*-th source can be written as

(4) $$g\left(\gamma_{k},\eta_{k}\right)=\left[\begin{array}{c} \sin\gamma_{k}e^{j\eta_{k}}\\ \cos\gamma_{k} \end{array}\right].$$

Hence, we can obtain a spatial and polarization joint steering vector as

(5) $$a\left(\theta_{k},r_{k},\gamma_{k},\eta_{k}\right)=g\left(\gamma_{k},\eta_{k}\right)⊗b\left(\theta_{k},r_{k}\right),$$

where ⊗ represents the Kronecker product, $a\left(\theta_{k},r_{k},\gamma_{k},\eta_{k}\right)$ is the $2\left(2L+1\right)\times 1$ vector. At time *t*, the signal data vector of the COLD array can be expressed as the following $2\left(2L+1\right)\times 1$ vector:

(6) $$\begin{array}{cc} x\left(t\right) & =\sum_{k=1}^{K}g\left(\gamma_{k},\eta_{k}\right)⊗b\left(\theta_{k},r_{k}\right)s_{k}\left(t\right)+n\left(t\right)\\ \; & =\left(G⊙B\right)s\left(t\right)+n\left(t\right)\\ \; & =\mathrm{As}\left(t\right)+n\left(t\right) \end{array},$$

where ⊙ represents the Khatri–Rao product, $A=\left[a\left(\theta_{1},r_{1},\gamma_{1},\eta_{1}\right),...,a\left(\theta_{K},r_{K},\gamma_{K},\eta_{K}\right)\right]$ signifies the $2\left(2L+1\right)\times K$ manifold matrix, $B=\left[b\left(\theta_{1},r_{1}\right),...,b\left(\theta_{K},r_{K}\right)\right]$ stands for spatial manifold matrix, $G=\left[g\left(\gamma_{1},\eta_{1}\right),...,g\left(\gamma_{K},\eta_{K}\right)\right]$ stands for the 2×K polarization manifold matrix, $s\left(t\right)={\left[s_{1}\left(t\right),...,s_{K}\left(t\right)\right]}^{T}$ represents the K×1 signal vector, and $n\left(t\right)$ represents the $2\left(2L+1\right)\times 1$ additive noise vector of COLD array with zero mean.

From (5), the signal data vector can be divided into horizontal and vertical components, expressed as

(7) $$x\left(t\right)=\left[\begin{array}{c} x_{1}\left(t\right)\\ x_{2}\left(t\right) \end{array}\right]=\left[\begin{array}{c} {\mathrm{BP}}_{1}\\ {\mathrm{BP}}_{2} \end{array}\right]s\left(t\right)+n\left(t\right),$$

where $x_{1}\left(t\right)$ is composed of the first 2L+1 rows of $x\left(t\right)$, $x_{1}\left(t\right)={\left[x_{1,-L}\left(t\right),...,x_{1,L}\left(t\right)\right]}^{T}$, and $x_{2}\left(t\right)$ containing the last 2L+1 rows, $x_{2}\left(t\right)={\left[x_{2,-L}\left(t\right),...,x_{2,L}\left(t\right)\right]}^{T}$. $P_{1}$ and $P_{2}$ are diagonal matrices, whose *k*-th diagonal elements are $\sin\gamma_{k}e^{j\eta_{k}}$ and $\cos\gamma_{k}$, respectively. Hence, the steering vector $a\left(\theta_{k},r_{k},\gamma_{k},\eta_{k}\right)$ can be rewritten as two components, i.e., $a\left(\theta_{k},r_{k},\gamma_{k},\eta_{k}\right)={\left[a_{1,-L,k},...,a_{1,L,k},a_{2,-L,k},...,a_{2,L,k}\right]}^{T}$, where $a_{1,l,k}=\sin\gamma_{k}e^{j\eta_{k}}b_{l,k}$ and $a_{2,l,k}=\cos\gamma_{k}b_{l,k}$.

## 3. The Proposed Method

According to the definition in [25], the FOC of $x_{p}\left(t\right)$, $x_{q}\left(t\right)$, $x_{i}\left(t\right)$, $x_{j}\left(t\right)$ is given by

(8) $$\begin{array}{cc} C\left(x_{p},x_{q}^{*},x_{i},x_{j}^{*}\right) & =E\{x_{p}x_{q}^{*}x_{i}x_{j}^{*}\}\\ \; & -E\left\{x_{p}x_{q}^{*}\right\}E\left\{x_{i}x_{j}^{*}\right\}\\ \; & -E\left\{x_{p}x_{i}\right\}E\left\{x_{q}^{*}x_{j}^{*}\right\}\\ \; & -E\left\{x_{p}x_{j}^{*}\right\}E\left\{x_{q}^{*}x_{i}\right\} \end{array}.$$

### 3.1. Spatial Manifold Matrix Estimation

Using the signal data vector, we can construct the FOC matrix of the *l*-th COLD antenna $C_{l}$ as follows [26]

(9) $$\begin{array}{cc} C_{l} & =\sum_{i=1}^{2}\mathrm{cum}\left(x_{i,l}\left(t\right),x_{i,0}^{*}\left(t\right),x\left(t\right),x^{H}\left(t\right)\right)\\ \; & =\sum_{i=1}^{2}\left\{\sum_{k=1}^{K}a_{i,l,k}a_{i,0,k}a_{k}a_{k}^{H}\times \mathrm{cum}\left(s_{k}\left(t\right),s_{k}^{*}\left(t\right),s_{k}\left(t\right),s_{k}^{*}\left(t\right)\right)\right\}\\ \; & =\sum_{k=1}^{K}b_{l,k}a_{k}a_{k}^{H}c_{4s_{k}} \end{array},$$

where ${\left(\cdot \right)}^{*}$ represents complex conjugate, ${\left(\cdot \right)}^{H}$ represents conjugate transpose, and the FOC matrix $C_{l}$ can be expressed in compact form as $C_{l}=A\Phi_{l}C_{4s}A^{H}$ with dimensions of $2\left(2L+1\right)\times 2\left(2L+1\right)$, $c_{4s_{k}}=\mathrm{cum}\left(s_{k}\left(t\right),s_{k}^{*}\left(t\right),s_{k}\left(t\right),s_{k}^{*}\left(t\right)\right)$ stands for the FOC of *k*-th signal, $\Phi_{l}$ is diagonal matrix, whose diagonal elements are related to the amplitude-phase corresponding of *l*-th COLD array, $\Phi_{l}=\mathrm{diag}\left[\rho_{l,1}e^{j\tau_{l,1}},...,\rho_{l,K}e^{j\tau_{l,K}}\right]$, $C_{4s}=\mathrm{diag}\left[c_{4s_{1}},...,c_{4s_{K}}\right]$.

From (9), for the complete COLD array, we can construct 2L+1 similar cumulant matrices by varying the sensor index. Subsequently, a long matrix Ξ can be obtained by cascading these cumulant matrices

(10) $$\Xi =\left[\begin{array}{c} C_{-L}\\ \vdots \\ C_{0}\\ \vdots \\ C_{L} \end{array}\right]=\left[\begin{array}{c} A\Phi_{-L}C_{4s}A^{H}\\ \vdots \\ A\Phi_{0}C_{4s}A^{H}\\ \vdots \\ A\Phi_{L}C_{4s}A^{H} \end{array}\right],$$

where $\Phi_{0}$ is the identity matrix. The covariance matrix of this joint long matrix is given by

(11) $$R=E\{\Xi \Xi^{H}\}.$$

The eigen-decomposition of the covariance matrix R yields

(12) $$R=U\Sigma U^{H}=U_{S}\Sigma_{S}U_{S}^{H}+U_{N}\Sigma_{N}U_{N}^{H},$$

where U contains the eigenvectors of R, Σ is the diagonal matrix, the signal subspace $U_{S}$ corresponds to the *K* largest eigenvalues with the dimensions $2{\left(2L+1\right)}^{2}\times K$, while the noise subspace $U_{N}$ corresponds to the $2{\left(2L+1\right)}^{2}-K$ smallest eigenvalues.

The signal subspace $U_{S}$ comprises the subspace associated with the cumulant matrices of all array elements, which can be partitioned into 2L+1 block sub-matrices as follows:

(13) $$U_{S}={\left[U_{S_{-L}}^{T},\cdots ,U_{S_{0}}^{T},\cdots ,U_{S_{L}}^{T}\right]}^{T},$$

where $U_{S_{0}}$, formed by the elements from rows $2\left(2L+1\right)L+1$ to $2\left(2L+1\right)L+1$ of $U_{S}$, represents the subspace corresponding to the cumulant matrix of the reference. Due to the rotation-invariant property between the reference cumulant matrix and other sensors’ matrices, there exists a full-rank matrix T satisfying

(14) $$U_{S}=\left[\begin{array}{c} A\Phi_{-L}\\ \vdots \\ A\Phi_{0}\\ \vdots \\ A\Phi_{L} \end{array}\right]T.$$

From (14), between $U_{S_{l}}$ and $U_{S_{0}}$, we can have the following relationship as

(15) $$U_{S_{l}}=U_{S_{0}}T^{-1}\Phi_{l}T=U_{S_{0}}\Psi_{l},$$

where $\Psi_{l}=T^{-1}\Phi_{l}T$, $\Phi_{l}$ is diagonal matrix, whose diagonal elements are the eigenvalues of $\Psi_{l}$. Using the least squares (LS), we can obtain the following formulation:

(16) $$\min{∥\Delta U∥}^{2}\;\;s.t.U_{S_{l}}+\Delta U=U_{S_{0}}\Psi_{l}.$$

From (16), we can derive

(17) $$\begin{array}{cc} \Psi_{l} & ={\left(U_{S_{0}}^{H}U_{S_{0}}\right)}^{-1}U_{S_{0}}^{H}U_{S_{l}}\\ \; & =U_{S_{0}}^{+}U_{S_{l}} \end{array},$$

where ${\left(\cdot \right)}^{+}$ represents pseudo-inverse. Obviously, ${\hat{\Phi }}_{l}$ can be estimated by performing the eigen-decomposition of $\Psi_{l}$. Iterate through different array elements and repeat the aforementioned steps (17), the spatial manifold matrix $\hat{B}$ can be estimated as follows:

(18) $$\hat{B}=\left[\begin{array}{c} {\mathrm{diag}}^{-1}\left({\hat{\Phi }}_{-L}\right)\\ \vdots \\ {\mathrm{diag}}^{-1}\left({\hat{\Phi }}_{0}\right)\\ \vdots \\ {\mathrm{diag}}^{-1}\left({\hat{\Phi }}_{L}\right) \end{array}\right]=\left[\hat{b}\left(\theta_{1},r_{1}\right),...,\hat{b}\left(\theta_{K},r_{K}\right)\right],$$

where ${\mathrm{diag}}^{-1}\left(\cdot \right)$ represents the row vector operation, and the estimation of spatial steering vector of *k*-th signal is $\hat{b}\left(\theta_{k},r_{k}\right)={\left[{\hat{b}}_{-L,k},...,{\hat{b}}_{0,k},...,{\hat{b}}_{L,k}\right]}^{T}$.

### 3.2. Polarization Parameters Estimation

From (7), obviously, the FOC matrix $C_{l}$ can be divided into

(19) $$C_{l}=\left[\begin{array}{c} B\Phi_{l}P_{1}C_{4s}B^{H}\\ B\Phi_{l}P_{2}C_{4s}B^{H} \end{array}\right],$$

where the first half of $C_{l}$ corresponds to the horizontal component, while the second half corresponds to the vertical component. Therefore, $U_{S_{l}}$ can be further divided into

(20) $$U_{S_{l}}={\left[U_{S_{l,1}}^{T},U_{S_{l,2}}^{T}\right]}^{T},$$

where $U_{S_{l,1}}$ is formed by the first 2L+1 rows of $U_{S_{l}}$ and $U_{S_{l,2}}$ formed by the last 2L+1 rows. Sub-matrices $U_{S_{-L,1}},...,U_{S_{L,1}}$ and $U_{S_{-L,2}},...,U_{S_{L,2}}$ can be extracted from the signal subspace $U_{S}$, the horizontal and vertical component subspaces, $E_{h}$ and $E_{v}$, can then be constructed as

(21) $$E_{h}=\left[\begin{array}{c} U_{S_{-L,1}}\\ \vdots \\ U_{S_{0,1}}\\ \vdots \\ U_{S_{L,1}} \end{array}\right],E_{v}=\left[\begin{array}{c} U_{S_{-L,2}}\\ \vdots \\ U_{S_{0,2}}\\ \vdots \\ U_{S_{L,2}} \end{array}\right].$$

By applying ESPRIT, we can rewrite (21) as

(22) $$E_{h}=\left[\begin{array}{c} B\Phi_{-L}P_{1}\\ \vdots \\ B\Phi_{L}P_{1} \end{array}\right]T,E_{v}=\left[\begin{array}{c} B\Phi_{-L}P_{2}\\ \vdots \\ B\Phi_{L}P_{2} \end{array}\right]T.$$

With component matrices $E_{h}$ and $E_{v}$, we can obtain the following matrix:

(23) $$\begin{array}{cc} D & =E_{h}^{+}E_{v}\\ \; & =T^{-1}P_{1}^{-1}P_{2}T=T^{-1}\mathrm{PT} \end{array}.$$

Substituting matrices $P_{1}$ and $P_{2}$ into (23), the polarized diagonal matrix P can be expressed as

(24) $$\begin{array}{c} P=\mathrm{diag}\left[\cot\gamma_{1}e^{-j\eta_{1}},...,\cot\gamma_{K}e^{-j\eta_{K}}\right] \end{array}.$$

Like (15), $\hat{P}$ can be estimated by performing eigen-decomposition of D. As a result, the polarization parameters estimates of *k*-th source are given by

(25) $$\begin{array}{cc} {\hat{\gamma }}_{k} & =\mathrm{arc}\cot\left|{\hat{P}}_{k}\right|\\ {\hat{\eta }}_{k} & =-\mathrm{angle}\left({\hat{P}}_{k}\right) \end{array},$$

where ${\hat{P}}_{k}$ represents the *k*-th diagonal element of $\hat{P}$.

### 3.3. Coarse Estimation

The angle(·) operator can extract the phase of ${\hat{b}}_{l,k}$ but introduces phase ambiguity when the spacing between two COLDs exceeds λ/2. To resolve this ambiguity, disambiguation operations utilizing the coarse reference estimates are employed to obtain the unambiguous phase ${\hat{\tau }}_{l,k}$ for fine estimation. Then, we impose structural constraints on the array configuration to obtain unambiguous reference values. Assuming there exist three sensors, whose positions are $d_{p}$, $d_{c}$, and $d_{q}$, respectively, the phase factor will be coarse but unambiguous, when the spacing of these three sensors satisfies $\left|d_{p}-d_{c}\right|⩽\lambda /2$ and $\left|d_{q}-d_{c}\right|⩽\lambda /2$. The relationship between these sensors can be established as

(26) $$\left\{\begin{array}{c} \sqrt{r_{k}^{2}+d_{c}^{2}-2r_{k}d_{c}\sin\alpha_{k}}-\sqrt{r_{k}^{2}+d_{p}^{2}-2r_{k}d_{p}\sin\alpha_{k}}=\frac{{\hat{\psi }}_{c,p,k}}{2\pi /\lambda }\\ \sqrt{r_{k}^{2}+d_{c}^{2}-2r_{k}d_{c}\sin\alpha_{k}}-\sqrt{r_{k}^{2}+d_{q}^{2}-2r_{k}d_{q}\sin\alpha_{k}}=\frac{{\hat{\psi }}_{c,q,k}}{2\pi /\lambda } \end{array}\right.,$$

where ${\hat{\psi }}_{c,p,k}=\mathrm{angle}\left(\hat{B}\left(p,k\right)/\hat{B}\left(c,k\right)\right)$ and ${\hat{\psi }}_{c,p,k}=\mathrm{angle}\left(\hat{B}\left(q,k\right)/\hat{B}\left(c,k\right)\right)$. In this work, $d_{c}$ represents the coordinates of the reference element, while $d_{p}=d_{-1}$ and $d_{q}=d_{1}$ denote the coordinates of its adjacent elements located at λ/2 on either side. Hence, from (18), the unambiguous phases of the *k*-th steering vector can be denoted as ${\hat{\tau }}_{-1,k}$ and ${\hat{\tau }}_{1,k}$. Then, the coarse estimates of $\theta_{k}$ and $r_{k}$ can be given by

(27) $$\left\{\begin{array}{cc} {\hat{r}}_{k}^{\left(c\right)} & =\frac{d_{1}{\left(\frac{{\hat{\tau }}_{-1,k}}{2\pi /\lambda }\right)}^{2}-d_{-1}{\left(\frac{{\hat{\tau }}_{1,k}}{2\pi /\lambda }\right)}^{2}+d_{-1}d_{1}^{2}-d_{1}d_{-1}^{2}}{2d_{1}\frac{{\hat{\tau }}_{-1,k}}{2\pi /\lambda }-2d_{-1}\frac{{\hat{\tau }}_{1,k}}{2\pi /\lambda }}\\ {\hat{\theta }}_{k}^{\left(c\right)} & =\mathrm{arc}\sin\left(\frac{2{\hat{r}}_{k}^{\left(c\right)}\frac{{\hat{\tau }}_{-1,k}}{2\pi /\lambda }-{\left(\frac{{\hat{\tau }}_{-1,k}}{2\pi /\lambda }\right)}^{2}+d_{-1}^{2}}{2{\hat{r}}_{k}^{\left(c\right)}d_{-1}}\right) \end{array}\right..$$

### 3.4. Fine Estimation

Let ${\hat{\phi }}_{l,k}$ denote the ambiguous phase. According to the (2), there exists an integer-multiplicity set $S_{l}$ relationship between ${\hat{\phi }}_{l,k}$ and ${\hat{\tau }}_{l,k}$, expressed as $S_{l}=\left\{\left.{\hat{\phi }}_{l,k}+2\pi n\right|\;\;n\in Z\right\}$. With (27), by using ${\hat{\theta }}_{k}^{\left(c\right)}$ and ${\hat{r}}_{k}^{\left(c\right)}$, we can construct the unambiguous phase reference values as follows:

(28) $${\hat{\tau }}_{l,k}^{co}=\frac{2\pi }{\lambda }\left({\hat{r}}_{k}^{\left(c\right)}-{\hat{r}}_{l,k}^{\left(c\right)}\right).$$

Thus, the exact estimation ${\hat{\tau }}_{l,k}$ is the value in $S_{l}$ that minimizes the deviation from the coarse estimate ${\hat{\tau }}_{l,k}^{co}$, i.e.,

(29) $${\hat{\tau }}_{l,k}=\underset{n}{\arg\min}\left|\tau_{l,k}^{co}-{\hat{\phi }}_{l,k}+2\pi n\right|.$$

Furthermore, the phase term satisfies the following relationship with respect to the angle and range:

(30) $$\frac{\tau_{l,k}}{2\pi /\lambda }=r_{k}-r_{l,k}=r_{k}-\sqrt{r_{k}^{2}+d_{l}^{2}-2r_{k}d_{l}\sin\theta_{k}}.$$

Substituting the ${\hat{\tau }}_{-L,k},\cdots ,{\hat{\tau }}_{l,k},\cdots ,{\hat{\tau }}_{L,k}$ into (30) and simplifying, we can construct coefficient matrices which consist of 2L equation relations

(31) $$V_{k}=\left[\begin{array}{cc} 2\frac{{\hat{\tau }}_{-L,k}}{2\pi /\lambda } & -2d_{-L}\\ \vdots & \vdots \\ 2\frac{{\hat{\tau }}_{L,k}}{2\pi /\lambda } & -2d_{L} \end{array}\right],W_{k}=\left[\begin{array}{c} {\left(\frac{{\hat{\tau }}_{-L,k}}{2\pi /\lambda }\right)}^{2}-d_{-L}^{2}\\ \vdots \\ {\left(\frac{{\hat{\tau }}_{L,k}}{2\pi /\lambda }\right)}^{2}-d_{L}^{2} \end{array}\right],$$

where the unknown vector to be solved is denoted as $\Delta_{f,k}^{T}={\left[r_{k}\;r_{k}\sin\theta_{k}\right]}^{T}$. These vectors can be further rewritten as a matrix equation $V_{k}\times \Delta_{f,k}=W_{k}$. By applying LS, the ${\hat{\Delta }}_{f,k}$ can be solved as

(32) $$\begin{array}{cc} {\hat{\Delta }}_{f,k} & ={\left(V_{k}^{H}V_{k}\right)}^{-1}V_{k}^{H}W_{k}\\ \; & =V_{k}^{+}W_{k} \end{array}.$$

Finally, the fine estimates of angle and range can be derived as

(33) $$\left\{\begin{array}{c} {\hat{r}}_{k}^{fi}={\hat{\Delta }}_{f,k}\left(1\right)\\ {\hat{\theta }}_{k}^{fi}=\mathrm{arc}\sin\left(\frac{{\hat{\Delta }}_{f,k}\left(2\right)}{{\hat{\Delta }}_{f,k}\left(1\right)}\right) \end{array}\right..$$

### 3.5. Asymptotic Variance for Joint Parameters

The theoretical phase at the *l*-th sensor from the *k*-th source is given by

(34) $$\vartheta_{l,k}=e^{j\frac{2\pi }{\lambda }\left(r_{k}-\sqrt{r_{k}^{2}+d_{l}^{2}-2r_{k}d_{l}\sin\theta_{k}}\right)},$$

while the corresponding polarization factor for the *k*-th source is

(35) $$p_{k}=\cot\gamma_{k}e^{-j\eta_{k}}.$$

Additionally, the estimate ${\hat{\vartheta }}_{l,k}$ of $\vartheta_{l,k}$, is the $\left(l,k\right)$-th element of $\hat{B}$, while ${\hat{p}}_{k}$ is obtained from the *k*-th diagonal element of $\hat{P}$. Thus, the deviations between the true and estimated values are $\delta \vartheta_{l,k}={\hat{\vartheta }}_{l,k}-\vartheta_{l,k}$ and $\delta p_{k}={\hat{p}}_{k}-p_{k}$ [13].

For simplicity, the fine estimates of angle and range are defined as ${\hat{\theta }}_{k}$ and ${\hat{r}}_{k}$, whose deviations are defined as $\delta \theta_{k}={\hat{\theta }}_{k}-\theta_{k}$ and $\delta r_{k}={\hat{r}}_{k}-r_{k}$. In addition, the deviations of polarization angle and polarization phase difference are defined as $\delta \gamma_{k}={\hat{\gamma }}_{k}-\gamma_{k}$ and $\delta \eta_{k}={\hat{\eta }}_{k}-\eta_{k}$, respectively.

Using the first-order Taylor expansion for $\vartheta_{l,k}$ and $p_{k}$, we can obtain

(36) $$\vartheta_{l,k}\approx {\hat{\vartheta }}_{l,k}+\left(\theta_{k}-{\hat{\theta }}_{k}\right)\frac{\partial \vartheta_{l,k}}{\partial \theta_{k}}+\left(r_{k}-{\hat{r}}_{k}\right)\frac{\partial \vartheta_{l,k}}{\partial r_{k}},$$

(37) $$p_{k}\approx {\hat{p}}_{k}+\left(\gamma_{k}-{\hat{\gamma }}_{k}\right)\frac{\partial p_{k}}{\partial \gamma_{k}}+\left(\eta_{k}-{\hat{\eta }}_{k}\right)\frac{\partial p_{k}}{\partial \eta_{k}},$$

where the partial derivatives of $\vartheta_{l,k}$ to ${\hat{\theta }}_{k}$ and ${\hat{r}}_{k}$ are

(38) $$\begin{array}{cc} \frac{\partial \vartheta_{l,k}}{\partial \theta_{k}} & =\vartheta_{l,k}j\frac{2\pi }{\lambda }\left(\frac{r_{k}d_{l}\cos\theta_{k}}{\sqrt{r_{k}^{2}+d_{l}^{2}-2r_{k}d_{l}\sin\theta_{k}}}\right),\\ \frac{\partial \vartheta_{l,k}}{\partial r_{k}} & =\vartheta_{l,k}j\frac{2\pi }{\lambda }\left(1-\frac{2r_{k}-2d_{l}\sin\theta_{k}}{2\sqrt{r_{k}^{2}+d_{l}^{2}-2r_{k}d_{l}\sin\theta_{k}}}\right), \end{array}$$

and

(39) $$\begin{array}{cc} \frac{\partial p_{k}}{\partial \gamma_{k}} & =-\frac{1}{\sin^{2}\gamma_{k}}e^{-j\eta_{k}},\\ \frac{\partial p_{k}}{\partial \eta_{k}} & =-j\cot\gamma_{k}e^{-j\eta_{k}}. \end{array}$$

Substituting (38) and (39) into (36) and (37), respectively, the final expansion yields

(40) $$j\frac{2\pi r_{k}d_{l}}{\lambda r_{l,k}}\cos\theta_{k}\vartheta_{l,k}\delta \theta_{k}+j\frac{2\pi \left(r_{l,k}-r_{k}+d_{l}\sin\theta_{k}\right)}{\lambda r_{l,k}}\vartheta_{l,k}\delta r_{k}=\delta \vartheta_{l,k},$$

(41) $$-\frac{1}{\sin^{2}\gamma_{k}}e^{-j\eta_{k}}\delta \gamma_{k}-j\cot\gamma_{k}e^{-j\eta_{k}}\delta \eta_{k}=\delta p_{k}.$$

With the same principle as (31), 2L equations can be listed, which can than be rewritten in matrix form

(42) $$Z_{k}=\left[\begin{array}{cc} j\upsilon_{-L,k}\vartheta_{-L,k} & j\omega_{-L,k}\vartheta_{-L,k}\\ \vdots & \vdots \\ j\upsilon_{L,k}\vartheta_{L,k} & j\omega_{L,k}\vartheta_{L,k} \end{array}\right],\Lambda_{k}=\left[\begin{array}{c} \delta \vartheta_{-L,k}\\ \vdots \\ \delta \vartheta_{L,k} \end{array}\right],$$

where

(43) $$\upsilon_{l,k}=\frac{2\pi r_{k}d_{l}}{\lambda r_{l,k}}\cos\theta_{k},\omega_{l,k}=\frac{2\pi \left(r_{l,k}-r_{k}+d_{l}\sin\theta_{k}\right)}{\lambda r_{l,k}},$$

and the unknown vector to be solved is $\Gamma_{k}={\left[\delta \theta_{k}\;\;\delta r_{k}\right]}^{T}$, which satisfies $Z_{k}\times \Gamma_{k}=\Lambda_{k}$. By applying LS, we have the following results:

(44) $$\Gamma_{k}={\left(Z_{k}^{H}Z_{k}\right)}^{-1}Z_{k}^{H}\Lambda_{k}=Z_{k}^{+}\Lambda_{k},$$

Thus, the closed-form variance expressions can be given by

(45) $$\begin{array}{cc} E\left\{{\left(\delta \theta_{k}\right)}^{2}\right\} & =E\left\{{\left[Z_{k}^{+}\Lambda_{k}{\left(Z_{k}^{+}\Lambda_{k}\right)}^{T}\right]}_{1}\right\},\\ E\left\{{\left(\delta r_{k}\right)}^{2}\right\} & =E\left\{{\left[Z_{k}^{+}\Lambda_{k}{\left(Z_{k}^{+}\Lambda_{k}\right)}^{T}\right]}_{2}\right\}. \end{array}$$

where ${\left[\cdot \right]}_{1}$ represents the first diagonal element, and ${\left[\cdot \right]}_{2}$ represents the second diagonal element.

According to (41), $\delta \gamma_{k}$ and $\delta \eta_{k}$ correspond to the real and imaginary parts of $\delta p_{k}$, respectively. By exploiting the conjugate symmetry, the closed-form expressions for $\delta \gamma_{k}$ and $\delta \eta_{k}$ are

(46) $$\begin{array}{cc} \delta \gamma_{k} & =\frac{\left(\delta p_{k}e^{j\eta_{k}}+\delta p_{k}^{*}e^{-j\eta_{k}}\right)\sin^{2}\gamma_{k}}{-2},\\ \delta \eta_{k} & =\frac{\delta p_{k}e^{j\eta_{k}}-\delta p_{k}^{*}e^{-j\eta_{k}}}{-2j\cot\gamma_{k}}, \end{array}$$

and the variances of $\delta \gamma_{k}$ and $\delta \eta_{k}$ can be given by

(47) $$\left\{\begin{array}{c} E\left\{{\left(\delta \gamma_{k}\right)}^{2}\right\}=\frac{E\left\{{\left(\delta p_{k}e^{j\eta_{k}}+\delta p_{k}^{*}e^{-j\eta_{k}}\right)}^{2}\right\}\sin^{4}\gamma_{k}}{4}\\ E\left\{{\left(\delta \eta_{k}\right)}^{2}\right\}=\frac{E\left\{{\left(\delta p_{k}e^{j\eta_{k}}-\delta p_{k}^{*}e^{-j\eta_{k}}\right)}^{2}\right\}}{-4\cot^{2}\gamma_{k}} \end{array}\right..$$

The steps of the proposed method are summarized in Table 1.

### 3.6. Computational Complexity

In this section, we analyze the computational complexity of the proposed method, focusing on (a) 2L+1 FOC matrix construction requires $O\left\{4\left(2L+1\right)\times {\left(2L+1\right)}^{2}N\right\}$ flops; (b) the complexity of computing the covariance matrix R requires $O\{8{\left(2L+1\right)}^{5}\}$; (c) the eigen-decomposition of the covariance matrix requires $O\{8{\left(2L+1\right)}^{6}\}$ multiplication operations; (d) the complexity of manifold matrix estimation requires $O\{4\left(2L+1\right)K^{2}+2K^{2}\}$; (e) the complexity in computing D is $O\{2\left(2L+1\right)K^{2}\}$. Overall, the proposed method is approximately $O\{4{\left(2L+1\right)}^{3}N+8{\left(2L+1\right)}^{5}+8{\left(2L+1\right)}^{6}+\left(12L+8\right)K^{2}\}$. The computational complexity of the proposed algorithm and that of other comparative methods are summarized in Table 2, where $n_{\theta }$, $n_{r}$, $n_{\gamma }$, $n_{\eta }$ represent the number of searches for DOA, range, polarization angle, and polarization phase difference. As shown in Table 2, the proposed method showed better efficiency than FR-RARE and G-ESPRIT without peak search, though requiring more operations than He’s method.

### 3.7. CRB Derivation

This section derives the closed-form deterministic Cramér–Rao Bound (CRB) expressions for jointly estimating the DOA, range, and polarization parameters under exact near-field polarization conditions. We define the real-valued vector $\varepsilon ={\left[\theta^{T}\;\;r^{T}\;\;\eta^{T}\;\;\gamma^{T}\right]}^{T}$, where $\theta ={\left[\theta_{1},\theta_{2},\cdots ,\theta_{K}\right]}^{T}$, $r={\left[r_{1},r_{2},\cdots ,r_{K}\right]}^{T}$, $\eta ={\left[\eta_{1},\eta_{2},\cdots ,\eta_{K}\right]}^{T}$ and $\gamma ={\left[\gamma_{1},\gamma_{2},\cdots ,\gamma_{K}\right]}^{T}$. Thus, the CRB for the parameters in ε is given by [24]

(48) $$\mathrm{CRB}\left(\varepsilon \right)=\frac{\sigma_{n}^{2}}{2N}{\left\{ℜ\left[\left(\Upsilon^{H}P_{\Theta }^{⊥}\Upsilon \right)⊕\left(1_{4}⊙1_{4}^{T}⊗R_{s}^{T}\right)\right]\right\}}^{-1}.$$

where $\Upsilon =\left[\Upsilon_{\theta },\Upsilon_{r},\Upsilon_{\gamma },\Upsilon_{\eta }\right]$, $\Upsilon_{\theta }=\left[\frac{\partial \Theta }{\partial \theta_{1}},\cdots ,\frac{\partial \Theta }{\partial \theta_{K}}\right]$, $\Upsilon_{r}=\left[\frac{\partial \Theta }{\partial r_{1}},\cdots ,\frac{\partial \Theta }{\partial r_{K}}\right]$, $\Upsilon_{\gamma }=\left[\frac{\partial \Theta }{\partial \gamma_{1}},\cdots ,\frac{\partial \Theta }{\partial \gamma_{K}}\right]$, $\Upsilon_{\eta }=\left[\frac{\partial \Theta }{\partial \eta_{1}},\cdots ,\frac{\partial \Theta }{\partial \eta_{K}}\right]$, $P_{\Theta }^{⊥}=I_{2\left(2L+1\right)}-\Theta {\left(\Theta^{H}\Theta \right)}^{-1}\Theta^{H}$.

## 4. Simulation Results

Simulation experiments were performed to evaluate the performance of the proposed method compared with the existing G-ESPRIT method [22], FR-RARE method [23], He’s method [24], and the CRB [24] for an exact NF polarization scenario.

### 4.1. Experiment 1: RMSE vs. SNR

In this section, a symmetric linear COLD array consisting of 9 antenna elements was considered, with uniform spacing λ/4. Two uncorrelated NF sources arrived at the COLD array, with parameters $\left(-15^{\circ },\;5.5\lambda ,\;20^{\circ },-20^{\circ }\right)$ and $\left(20^{\circ },\;6\lambda ,\;30^{\circ },\;40^{\circ }\right)$ and the number of snapshots was set to N=2000. The root-mean-square error (RMSE) was utilized to evaluate the performance, defined as

(49) $$\mathrm{RMSE}=\sqrt{\frac{1}{500K}\sum_{k=1}^{K}\sum_{i=1}^{500}{\left(x_{k}-{\hat{x}}_{i,k}\right)}^{2}},$$

where $x_{k}$ stands for the true value of parameters $\theta_{k}$, $r_{k}$, $\gamma_{k}$, $\eta_{k}$ and ${\hat{x}}_{i,k}$ represents the estimate of the ${\hat{\theta }}_{i,k}$, ${\hat{r}}_{i,k}$, ${\hat{\gamma }}_{i,k}$, ${\hat{\eta }}_{i,k}$ at the *i*-th run in 500 Monte-Carlo trials.

**Figure 2:**
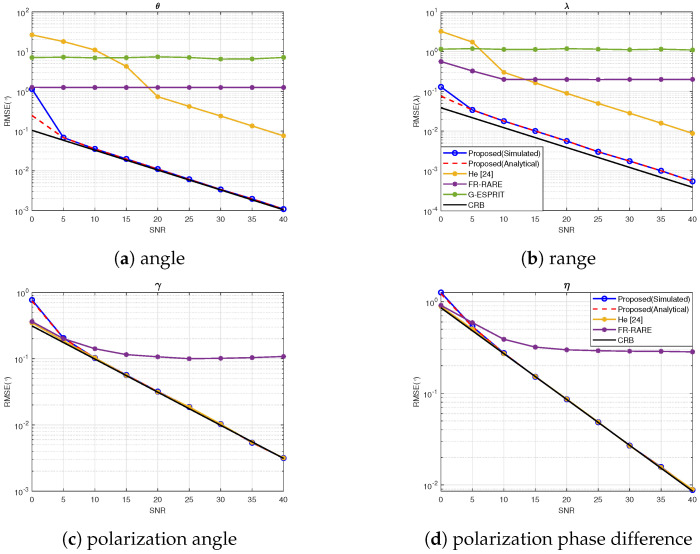
RMSE vs. SNR.

Figure 2 shows the RMSE curves of the spatial and polarization parameters, when the SNR varied from 0 dB to 40 dB. It can be seen that the estimation performance of both the proposed algorithm and He’s method improved as the SNR increased. As an amplitude-attenuation based method, He’s method exhibited limited estimation accuracy, suffering from mismatch under the low SNR. Among all the algorithms, the proposed algorithm more closely approximated the CRB and could exploit the whole aperture. In addition, due to the model approximation, G-ESPRIT and FR-RARE showed performance “saturation”.

### 4.2. Experiment 2: Scatter for Spatial and Polarization

**Figure 3:**
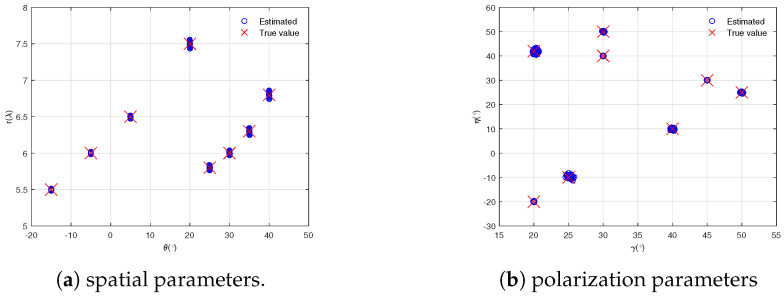
Scatter plots of 4-D parameters estimation.

In this section, we derived the maximum number of identifiable NF sources. Based on an analysis of the rank of the manifold matrix, the theoretical limit was established when the matrix satisfied the full-rank condition. Consider a 9-element COLD array with antenna positions $\left[-2,-1.6,-1.1,-0.5,0,0.5,1.3,1.5,2.2\right]$. Assume that 8 uncorrelated NF sources arrive at the array with locations $\left(-15^{\circ },5.5\lambda ,20^{\circ },-20^{\circ }\right)$, $\left(-5^{\circ },6\lambda ,30^{\circ },40^{\circ }\right)$, $\left(5^{\circ },6.5\lambda ,45^{\circ },30^{\circ }\right)$, $\left(20^{\circ },7.5\lambda ,50^{\circ },25^{\circ }\right)$, $\left(25^{\circ },5.8\lambda ,25^{\circ },-10^{\circ }\right)$, $\left(30^{\circ },6\lambda ,20^{\circ },42^{\circ }\right)$, $\left(35^{\circ },6.3\lambda ,40^{\circ },10^{\circ }\right)$, and $\left(40^{\circ },6.8\lambda ,30^{\circ },50^{\circ }\right)$. With the SNR fixed at 25 dB and the number of snapshots be 2000, Figure 3 presents scatter plots of joint parameters in a 2-D plane based on 500 Monte Carlo runs. As shown in Figure 3, the 4-D parameters were all closely distributed near the true values, which were automatically paired.

### 4.3. Experiment 3: RMSE vs. Path Loss Exponent

**Figure 4:**
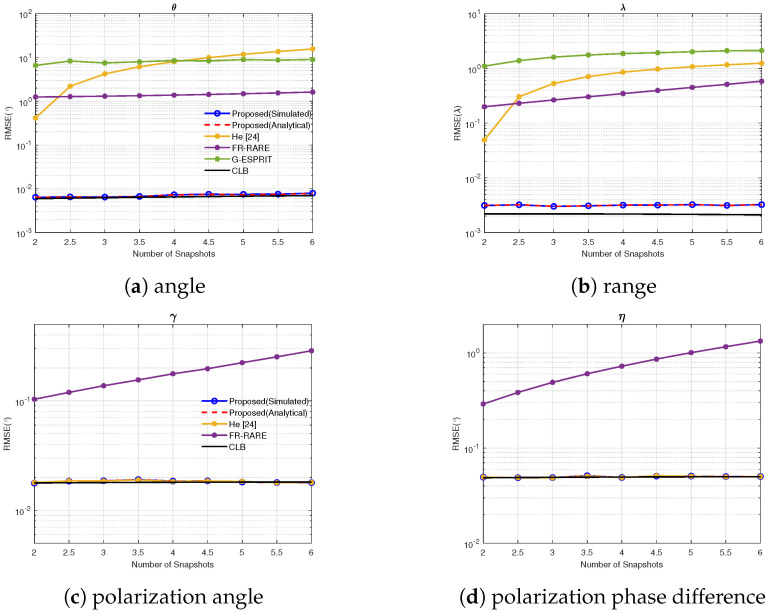
RMSE vs. path loss exponent.

In this section, we tested the algorithm performance with difference path loss exponents. The location of the sources and other parameters were set the same as in Experiment 1, and the path loss exponent varied from 2 to 6. As shown in Figure 4, the RMSE was approximately constant with the exponential increase in path loss. The reasons for the performance saturation of He’s method, G-ESPRIT, and FR-RARE were the same as in Experiment 1.

### 4.4. Experiment 4: Detection Capability for Closely Spaced Targets

**Figure 5:**
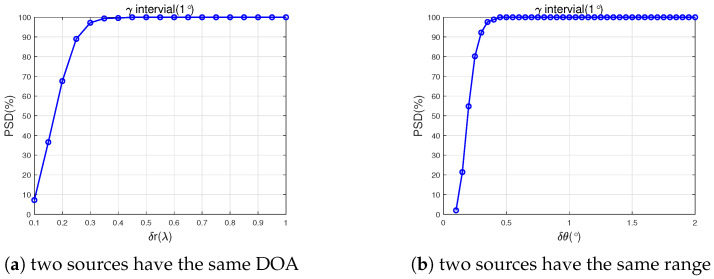
PSD vs. parameter interval.

In this section, we considered a scenario where two closely spaced impinging signals had the same DOA or same range. In Figure 5a, two sources had the same DOA parameterized by $\left(20^{\circ },5.5\lambda ,20^{\circ },-20^{\circ }\right)$, and $\left(20^{\circ },5.5\lambda +\delta \lambda ,30^{\circ },40^{\circ }\right)$, in Figure 5b, the two sources had the same range parameterized by $\left(20^{\circ },5.5\lambda ,20^{\circ },-20^{\circ }\right)$, and $\left(20^{\circ }+\delta \theta ,5.5\lambda ,30^{\circ },40^{\circ }\right)$, and the other parameters were set as same as Experiment 2. Successful detection was defined as a polarization angle RSME between the estimated $\hat{\gamma }$ and true value γ less than $1^{\circ }$. The probability of successful detection (PSD) was defined as a successful detection trial under 500 Monte-Carlo runs. As shown in Figure 5, when the DOA interval was larger than $0.5^{\circ }$ or the range interval was larger than 0.4λ, the proposed method offered 100% PSD for polarization angle; that is, we could refer to polarization information to distinguish two closely spaced impinging signals.

### 4.5. Experiment 5: RMSE vs. Correlation Coefficient

**Figure 6:**
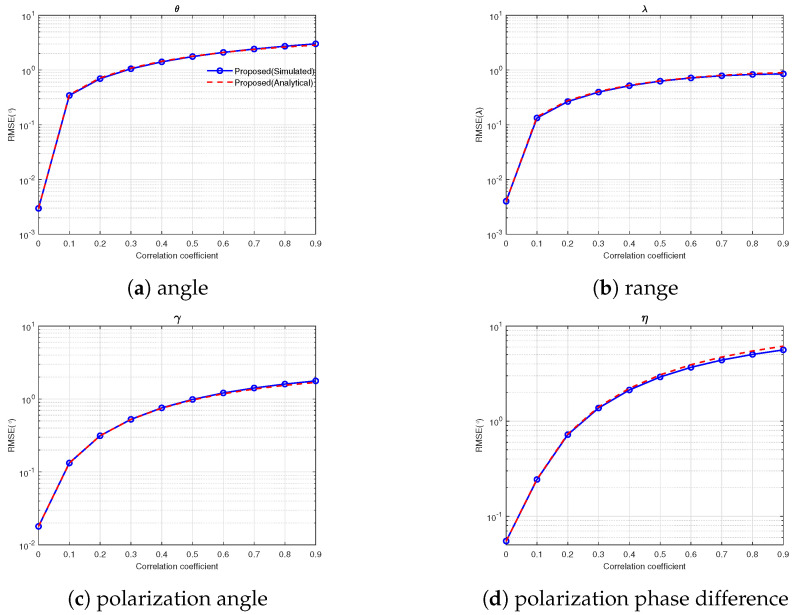
RMSE vs. correlation coefficient.

Assume that there are two correlated sources parameterized by $\left(10^{\circ },5.5\lambda ,15^{\circ },45^{\circ }\right)$ and $\left(22^{\circ },5.8\lambda ,35^{\circ },90^{\circ }\right)$, the other parameters were set the same as in Experiment 2, where correlation coefficient varied from 0.1 to 0.9. As can be seen from Figure 6, the RMSE of all spatial and polarization parameters increased with the increase in the correlation coefficient.

### 4.6. Experiment 6: RMSE vs. Snapshots

**Figure 7:**
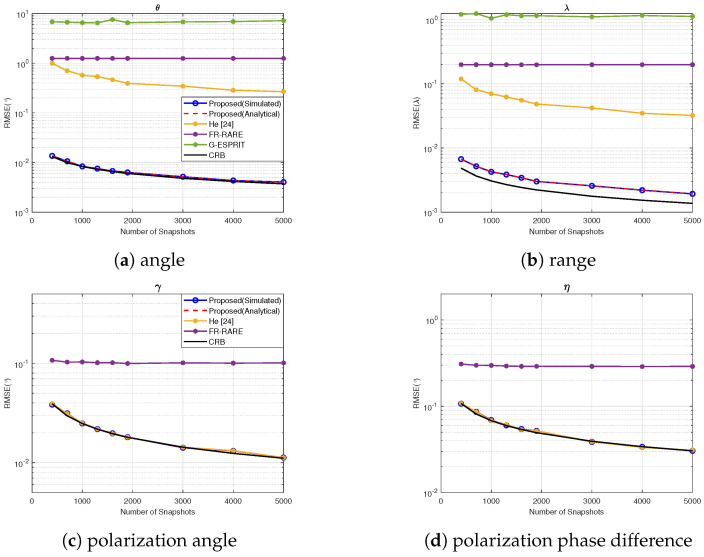
RMSE vs. the number of snapshots.

In this experiment, we evaluated the performance by varying the number of snapshots from 400 to 5000, the SNR was fixed as 25 dB, with the same source parameters as in Experiment 1. The RMSE of the 4-D parameters vs. the number of snapshots is shown in Figure 7. The results demonstrate that the RMSE of both the proposed algorithm and He’s method decreased with increasing snapshots, while our method had a better performance. In contrast, G-ESPRIT and FR-RARE were always in a performance saturation state.

## 5. Conclusions

The primary contribution of this paper is source localization under an exact NF polarization scenario. With a COLD array, a multiple-cumulant-matrices-based method was proposed for joint 4-D parameter estimation, formulated via least squares principles, with neither approximation nor aperture loss. After that, the asymptotic variances of 4-D parameters were derived. Simulation results demonstrated that the algorithm had superior performance under the exact NF polarization scenario.

## Figures and Tables

**Table 1:** Algorithmic steps.

Step	Operation
**1**	Construct $C_{-L},\cdots ,C_{0},\cdots ,C_{L}$ via (9) and the long matrix Ξ
	can be obtain via (10).
**2**	Compute the covariance matrix R and perform eigen-decomposition
	to obtain signal subspace $U_{S}$.
**3**	Using the LS and eigen-decomposition, we can obtain
	${\hat{\Phi }}_{-L},\cdots ,{\hat{\Phi }}_{0},\cdots ,{\hat{\Phi }}_{L}$ to construct $\hat{B}$.
**4**	Extract $U_{S_{-L,1}},\cdots ,U_{S_{L,1}}$ and $U_{S_{-L,2}},\cdots ,U_{S_{L,2}}$ from $U_{S}$ to construct $E_{h}$ and $E_{v}$.
	Matrix P is obtained by performing eigen-decomposition on matrix D,
	where matrix D can be obtain from (33). From the diagonal element of P,
	we have the estimates ${\hat{\gamma }}_{1},\cdots ,{\hat{\gamma }}_{k},\cdots ,{\hat{\gamma }}_{K}$ and ${\hat{\eta }}_{1},\cdots ,{\hat{\eta }}_{k},\cdots ,{\hat{\eta }}_{K}$.
**5**	**for** $k\in \left[1,K\right]$ **do**
	(1) The closed-form coarse estimation ${\hat{r}}_{k}^{\left(c\right)}$ and ${\hat{\theta }}_{k}^{\left(c\right)}$ can be obtained via (26) and (27).
	(2) Via (28) and (29), we can disambiguate the phase to construct matrices
	$V_{k}$ and $W_{k}$.
	(3) Based on LS method, the fine estimation ${\hat{r}}_{k}^{fi}$ and ${\hat{\theta }}_{k}^{fi}$ can be obtained by
	solving (33).
	**End for**

**Table 2:** Computational complexity of all compared algorithms.

Method	Complexity
Proposed	$O\{4{\left(2L+1\right)}^{3}N+8{\left(2L+1\right)}^{5}+8{\left(2L+1\right)}^{6}+\left(12L+8\right)K^{2}\}$
FR-RARE [22]	$O\{4{\left(2L+1\right)}^{2}N+8{\left(2L+1\right)}^{3}+4\left(L+1\right)\left(4L+2-K\right)\left(2L+1\right)Kn_{\theta }$
	$+\left(2L+1\right)\left(32L+16-4K+8\right)Kn_{r}+2\left(2L+1\right)\left(4L+2-K\right)n_{\gamma }n_{\eta }\}$
G-ESPRIT [23]	$O\{4{\left(2L+1\right)}^{2}N+8{\left(2L+1\right)}^{3}+32LK^{2}n_{\theta }+4\left(2L+1\right)\left(4L+2-K\right)n_{r}\}$
He [24]	$O\{4{\left(2L+1\right)}^{2}N+8{\left(2L+1\right)}^{3}+4{\left(2L+1\right)}^{2}K+2\left(2L+1\right)K^{2}\}$

## Data Availability

The data used to support the findings of this study are available from the corresponding author upon request.
